# The impact of Rituximab administered before transplantation in patients undergoing allogeneic hematopoietic stem cell transplantation: A real-world study

**DOI:** 10.3389/fimmu.2022.967026

**Published:** 2022-08-31

**Authors:** Xiya Wei, Yiyu Xie, Ruoyu Jiang, Huiyu Li, Heqing Wu, Yuqi Zhang, Ling Li, Shiyuan Zhou, Xiao Ma, Zaixiang Tang, Jun He, Depei Wu, Xiaojin Wu

**Affiliations:** ^1^ National Clinical Research Center for Hematologic Diseases, Jiangsu Institute of Hematology, The First Affiliated Hospital of Soochow University, Suzhou, China; ^2^ Institute of Blood and Marrow Transplantation, Collaborative Innovation Center of Hematology, Soochow University, Suzhou, China; ^3^ Department of Internal Medicine, Yale-New Haven Health/Bridgeport Hospital, Bridgeport, CT, United States; ^4^ Department of Epidemiology and Statistics, School of Public Health, Faculty of Medicine, Soochow University, Suzhou, China

**Keywords:** rituximab, B cell, prior to transplantation, allogeneic hematopoietic stem cell transplantation, EBV - Epstein-Barr virus, aGVHD: acute graft vs host disease

## Abstract

Rituximab is used to eliminate B cells as a chimeric monoclonal antibody directed against CD20, a B-cell antigen expressed on B cells. To explore the impact of rituximab administered before transplantation, we implemented a retrospective, monocentric study and utilized real-world data collected at our center between January 2018 and December 2020, and then followed until December 2021. Based on whether a dose of 375mg/m^2^ rituximab was used at least once within two weeks before transplantation, patients undergoing allo-HSCT were classified into two groups: rituximab (N=176) and non-rituximab (N=344) group. Amongst all the patients, the application of rituximab decreased EBV reactivation (P<0.01) and rituximab was an independent factor in the prevention of EBV reactivation by both univariate and multivariate analyses (HR 0.56, 95%CI 0.33-0.97, P=0.04). In AML patients, there were significant differences in the cumulative incidence of aGVHD between the two groups (P=0.04). Our data showed that rituximab was association with a decreased incidence of aGVHD in AML patients according to both univariate and multivariate analyses. There was no difference between the two groups in other sets of populations. Thus, our study indicated that rituximab administered before transplantation may help prevent EBV reactivation in all allo-HSCT patients, as well as prevent aGVHD in AML patients after allo-HSCT.

## Introduction

Rituximab is a monoclonal antibody against CD20, a protein found on the cell membranes of B lymphocytes before they terminally differentiate into plasma cells (1–3). CD20 is a hydrophobic transmembrane protein expressed on pre-B and mature B lymphocytes and a unique molecule that is resistant to internalization (4) and the portions that bind to rituximab are exposed to the extracellular environment (5–7). CD20 is responsible for regulating cell cycling. Rituximab activates complement efficiently through translocating CD20 to lipid rafts in the target cell membrane (8). These features might have contributed to the efficiency of killing by humoral and cellular effectors (4). Rituximab at a dose of 375 mg/m^2^ eliminates circulating B lymphocytes for 6 to 12 months after a cycle of infusions (3). This abrogates antibody-mediated immune responses, such as complement-mediated cytotoxicity, antibody-dependent cell-mediated cytotoxicity, and apoptosis (4).

Traditionally, rituximab has been used to treat lymphoma and autoimmune diseases (3, 5, 9, 10). Later, it has also been approved for remission induction and maintenance therapy of antineutrophil cytoplasmic antibodies (ANCA) associated vasculitis (AAV) and treatment of rheumatoid arthritis (RA), the most prevalent of systemic autoimmune rheumatic diseases (SARDs) (1, 11–13). To some extent, rituximab may play a role in the elimination of B cells on presence of anti- human leukocyte antigen (anti- HLA) antibodies. Inhibition of antibody production by using rituximab was found to desensitize patients with donor-specific antibodies (DSA) (14, 15). More recently, it was applied as an immunomodulatory agent in renal transplantation due to the setting of desensitization for patients that experience antibody-mediated rejection (AMR) (3, 16, 17).

Moreover, rituximab has been administered in allo-HSCT patients in several settings for its elimination of B cells. Until now, rituximab has also been used for the treatment of post-transplant lymphoproliferative disease (PTLD), prevention and treatment of graft versus host disease (GVHD) (18), ABO-incompatible transplantation, and desensitization in HLA-sensitized patients (18–24).

It was reported that rituximab induced the CD20+ B cells to a substantial reduction in circulating for up to 6 months through a cycle of infusions (25). Considering its long-term effects, it has been hypothesized that administration of rituximab before transplantation might impact patient outcomes following allo-HSCT (26). We currently knew very little regarding the impact of rituximab administered before transplantation in patients undergoing allo-HSCT. Therefore, we retrospectively compared the clinical outcomes in patients treated with or without rituximab to explore the impact of rituximab on allo-HSCT in a real-world setting.

## Patients and methods

### Study design

We implemented a retrospective, monocentric study and utilized real-world data collected at Soochow University. Consecutive patients (N=520) who received allo-HSCT were enrolled in the study from January 2016 to December 2020. Patients were then followed until December 2021. All patients provided written informed consent before the start of this study in accordance with the Declaration of Helsinki and the approval from the Faculty Hospital Ethics Committee at the First Affiliated Hospital of Soochow University. We divided the patients into two groups based on the application of rituximab: rituximab and non-rituximab group. At least two weeks prior to transplantation, 375mg/m^2^ of rituximab was used at least once in the rituximab group. Of these, all patients had prospective anti-HLA Ab testing performed. Sex, age, underlying disease, condition before transplantation (Complete remission (CR) vs non-CR), types of transplantation, stem cell source, conditioning regimens, donor-recipient sex match, blood type of donor to receipt, presence of anti-HLA antibody, the usage of anti-thymocyte globulin (ATG) and the infused number of each cell type in allografts such as mononuclear cells (MNC), CD3+ cells and CD4+ cells. The engraftment time to neutrophils or platelets was recorded. Transplant outcomes were measured in terms of acute GVHD (aGVHD), chronic GVHD (cGVHD), Epstein-Barr virus (EBV), cytomegalovirus (CMV), transplantation-associated thrombotic microangiopathy (TA-TMA), veno-occlusive disease (VOD), non-relapse mortality (NRM), relapse, progression-free survival (PFS), and overall survival (OS).

### Conditioning, GVHD prophylaxis, and desensitization regimens

Patients’ therapy followed our standard transplant protocols which included a conventional conditioning regimen for most patients based on BuCy/TBI-Cy or reduced intensity conditioning based on a combination of fludarabine and cyclophosphamide (27). Patients with a haplo-donor, unrelated donor (URD), or aplastic anemia (AA) were treated with anti-thymoglobulin, an *in vivo* T-cell depletion medication, as part of their conditioning regimen and these factors considered to be at high-risk for EBV reactivation (28). All patients received antiviral prophylaxis with intravenous acyclovir or oral valacyclovir at the start of conditioning. In patients with anti-HLA antibodies, desensitization regimens were implemented as instructed (29).

### Definitions and evaluations

The definition of neutrophil recovery was an absolute neutrophil count (ANC) >0.5×10^9^/L for 3 consecutive days (30). Platelet (PLT) recovery was defined as the count of PLT >20 × 10^9^/L for 7 consecutive days without platelet infusion. The diagnosis and grading of acute and chronic GVHD were based on the standard criteria and recorded by the clinicians. Bloodstream infections (BSIs) were defined as the isolation of bacteria from at least one blood culture (31). CMV or EBV reactivation was defined as a CMV DNA viral load of 100 copies/mL or an EBV DNA viral load of 100 copies/mL at any time after HSCT for at least one measurement (31). Relapse was defined by morphological evidence of disease in the peripheral blood, bone marrow, or extramedullary sites. Patients exhibiting minimal residual disease (for example, the presence of BCR/ABL RNA transcripts by PCR) were not classified as having relapsed. non-relapse mortality (NRM) was defined as death without preceding relapse, and OS referred to patients who survived until the final follow-up time point (31). PFS was defined as the length of time during and after the treatment of a disease, such as cancer, that a patient lives with the disease but the disease does not progress.

### Statistical analysis

Descriptive statistics, including frequency (percentage) for categorical variables and median (range) for quantitative variables, were used to summarize patient characteristics. BSIs were compared by means of the χ-squared or Fisher’s exact test, as appropriate. The primary endpoints were the recovery of neutrophils and platelets. Secondary endpoints were incidences of infection (EBV, CMV), aGVHD, cGVHD, relapse, NRM, PFS, and OS. Cumulative incidence was used to estimate the endpoints of infections, aGVHD and cGVHD to adjust for competing risks. For cGVHD, we considered relapse and death as competing events. The probabilities of PFS and OS were calculated using the Kaplan–Meier method. Potential prognostic factors were evaluated in univariate analyses by the log-rank test, with a P value <0.05 considered significant. Cox proportional hazards regression was used to identify risk factors associated with outcomes. Multivariate analyses were done for predefined subgroups. The assumption of proportional hazards for each factor in the Cox model was tested. The test indicated that the proportionality assumptions hold.

## Results

### Overall patient characteristics

There were 176 patients in the rituximab group and 344 patients in the non-rituximab group. The baseline characteristics of patients for the two groups are listed in **Table 1**. Statistically different characteristics between patients in the rituximab group compared with patients in the non-rituximab group included gender (P<0.01) and whether they had anti-HLA antibodies (P<0.01). The distribution of underlying diseases was also significantly different between the two groups (P<0.01).

**Table 1:** Baseline characteristics of the entire cohort.

Characteristics		No Rituximab	Rituximab	P-value
		N=344	N=176	
Gender	Female	176 (33.8)	131 (25.2)	<0.01
(N,%)	Male	168 (32.3)	45 (86.7)	
Age	Median	40	38	0.42
	Range	(6-68)	(4-67)	
Primary Diseases	AA	55 (10.6)	30 (5.8)	<0.01
(N,%)	ALL	75 (14.4)	26 (5.0)	
	AML	147 (28.3)	78 (15.0)	
	MDS	67 (12.9)	31 (6.0)	
	Other	0 (0.0)	11 (2.1)	
anti-HLA Antibodies	Negative	220 (42.3)	35 (6.7)	<0.01
(N,%)	Positive	124 (23.8)	141 (27.1)	
Stem Cell Source	BM	10 (1.9)	3 (0.6)	0.23
(N,%)	BM+PB	73 (14.0)	45 (8.7)	
	BM+PB+UCB	48 (9.2)	36 (6.9)	
	BM+UCB	3 (0.6)	2 (0.4)	
	PB	143 (27.5)	62 (11.9)	
	PB+UCB	67 (12.9)	28 (5.4)	
MNC	Median	11.9	11.4	0.20
	Range	(1.72-46.8)	(1.75-46.70)	
CD34+ cells	Median	4.41	4.08	<0.01
	Range	(1.11-12.10)	(0.68-43.70)	
CD3+ cells	Median	1.50	1.50	0.39
	Range	(0.13-6.44)	(0.25-7.31)	
CD4+ cells	Median	0.58	0.59	0.23
	Range	(0.04-2.68)	(0.08-3.03)	
CD8+ cells	Median	0.37	0.39	0.61
	Range	(0.03-1.51)	(0.05-2.25)	
NK cells	Median	2.06	2.06	0.10
	Range	(0.09-12.9)	(0.30-10.30)	
CD19+ cells	Median	3.57	3.60	0.20
	Range	(0.12-16.90)	(0.40-14.00)	
CR	No	88 (16.9)	59 (11.3)	0.06
(N,%)	Yes	256 (49.2)	117 (22.5)	
Conditioning	MAC	324 (62.3)	163 (31.3)	0.83
(N,%)	NMA	11 (2.1)	7 (1.3)	
	RIC	7 (1.3)	4 (0.8)	
	RTC	2 (0.4)	2 (0.4)	
ATG	No	171 (32.9)	88 (16.9)	1.00
(N,%)	Yes	173 (33.3)	88 (16.9)	
HLA disparity	Match	109 (21.0)	51 (9.8)	0.55
(N,%)	MisMatch	235 (45.2)	125 (24.0)	
Donor	Haplo	230 (44.2)	124 (23.8)	0.45
(N,%)	MisMUD	5 (1.0)	0 (0.0)	
	MRD	75 (14.4)	37 (7.1)	
	MUD	34 (6.5)	15 (28.8)	
Donor Recipient Gender	Match	187 (36.0)	81 (15.6)	0.08
(N,%)	MisMatch	157 (30.2)	95 (18.3)	
Female to Male	No	300 (57.7)	160 (30.8)	0.25
(N,%)	Yes	44 (8.5)	16 (3.1)	
ABO	Match	185 (35.6)	109 (21.0)	0.09
(N,%)	MisMatch	159 (30.6)	67 (12.9)	

Considering the significant difference between the two groups and the administration of rituximab in allo-HSCT, we next compared the impact of rituximab after allo-HSCT in these sets of populations.

### Engraftment

**Figure 1:**
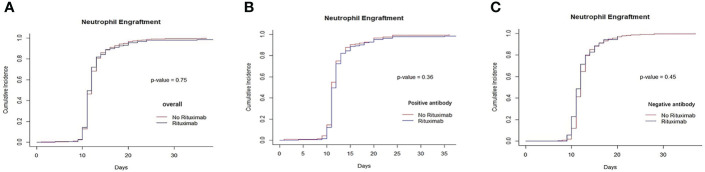
Recovery of neutrophils between rituximab and non-rituximab groups. **(A)** The cumulative incidence of neutrophil engraftment in entire patient cohort. There was no difference in the cumulative incidence of neutrophil engraftment rituximab and non- rituximab groups in entire patient cohort (P= 0.75). **(B)** The cumulative incidence of neutrophil engraftment in positive anti-HLA antibody group. There was no difference in the cumulative incidence of neutrophil engraftment between 2 groups in positive antibody group (P= 0.36). **(C)** The cumulative incidence of neutrophil engraftment in patients with negative anti-HLA antibody group. There was not different between 2 groups in the cumulative incidence of neutrophil engraftment in patients with negative anti- HLA antibody (P= 0.45).

**Figure 2:**
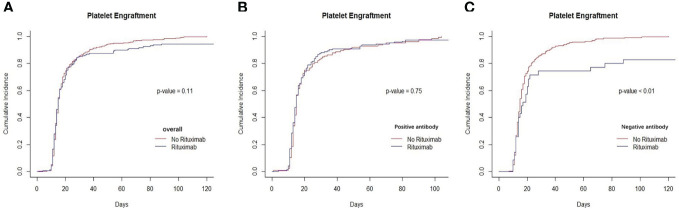
Recovery of platelets between rituximab and non-rituximab groups. **(A)** The cumulative incidence of platelets engraftment in entire patient cohort. There was no difference in the cumulative incidence of platelets engraftment rituximab and non- rituximab groups in entire patient cohort (P= 0.11). **(B)** The cumulative incidence of platelet engraftment in positive anti-HLA antibody group. There was no difference in the cumulative incidence of neutrophil engraftment between 2 groups in positive antibody group (P= 0.75). **(C)** The cumulative incidence of platelet engraftment in patients with negative anti-HLA antibody group. There was significantly between 2 groups in the cumulative incidence of platelet engraftment in patients with negative anti- HLA antibody (P,0.01).

There was not different in the cumulative incidence of neutrophil engraftment between the rituximab and non-rituximab groups across all sets of groups based on the presence of anti-HLA antibodies and different underlying diseases (**Figure 1** and **Supplementary Figure S1**). There was also no difference in the cumulative incidence of platelet engraftment between the rituximab and non-rituximab groups across the entire patient cohort (P=0.11, **Figure 2A**). Regarding underlying diseases, there were also no differences in the cumulative incidence of platelet engraftment between the rituximab and non-rituximab groups (**Supplementary Figure S2**). We found that the application of rituximab delayed the recovery of platelets in the negative anti-HLA antibodies group (P<0.01, **Figure 2C**), while not significantly impacting the positive anti-HLA antibodies group (P=0.75, **Figure 2B**).

### Infection complications

**Figure 3:**
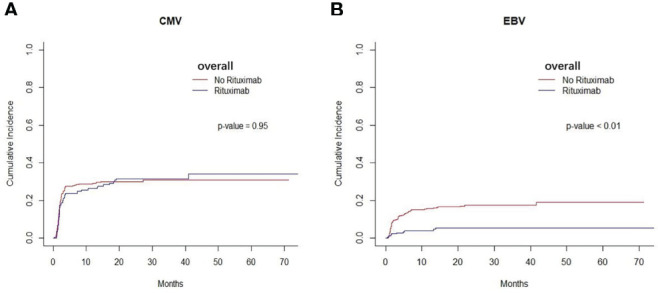
Infection of the entire cohort between two groups. **(A)** The cumulative incidence of CMV in entire patient cohort. There was no difference in the cumulative incidence of CMV between rituximab and non- rituximab groups in entire patient cohort (P= 0.95). **(B)** The cumulative incidence of EBV in entire patient cohort. There was comparable in the cumulative incidence of EBV between 2 groups in entire patient cohort (P < 0.01).

We found that the cumulative incidence of CMV occurred in 37.7% in non-rituximab group and 34.5% in rituximab group across the entire cohort. There was no difference in the cumulative incidence of CMV between non-rituximab and rituximab across the entire cohort (P=0.95, **Figure 3A**), so as other sets of populations (**Supplementary Figure S3**). The cumulative incidence of EBV reactivation was 21.6% in the non-rituximab group and 5.3% in the rituximab group across the entire cohort (**Table 2**). For the entire patient cohort, addition of rituximab decreased the incidence of EBV reactivation after allo-HSCT (P<0.01, **Figure 3B**). There was no difference in the occurrence of BSIs between the two groups (P=0.54).

**Table 2:** Outcomes of the entire patient cohort.

	Non-rituximab				Rituximab				P value
Time	Day 100	1 year	3 years	5 years	Day 100	1 year	3 years	5 years	
OS(%)	87.8	76.7	73.1	73.1	90.9	81.1	77.2	77.2	0.31
	(84.4-91.3)	(72.4-81.3)	(68.3-78.1)	(68.3-78.1)	(86.8-95.3)	(75.5-87.1)	(71.0-84.0)	(71.0-84.0)	
Relapse(%)	3.4	9.6	12.0	9.2	0.6	6.0	9.2	9.2	0.33
	(1.4-5.4)	(6.2-12.8)	(8.1-15.8)	(3.8-14.2)	(0.0-1.8)	(2.1-9.7)	(3.8-14.2)	(3.8-14.2)	
aGVHD(%)	41.1	41.1	41.1	41.1	34.4	34.4	34.4	34.4	0.20
	(35.6-46.3)	(35.6-46.3)	(35.6-46.3)	(35.6-46.3)	(26.8-41.2)	(26.8-41.2)	(26.8-41.2)	(26.8-41.2)	
cGVHD(%)	0.3	23.0	31.8	35.3	1.9	26.2	31.5	35.3	0.68
	(0.0-1.0)	(17.9-27.8)	(25.8-37.3)	(23.9-45.0)	(0.0-4.0)	(18.6-33.1)	(22.9-39.1)	(23.9-45.0)	
EBV(%)	10.9	17.2	19.5	21.6	3.0	3.7	5.3	5.3	<0.01
	(7.4-14.3)	(12.9-21.4)	(14.8-24.0)	(14.3-17.3)	(0.4-5.6)	(0.7-6.5)	(1.6-8.9)	(1.6-8.9)	
CMV(%)	27.9	32.0	34.1	37.7	22.9	28.3	34.5	37.7	0.95
	(22.7-32.7)	(26.6-37.0)	(28.4-39.3)	(27.4-46.5)	(16.2-29.0)	(21.0-34.9)	(26.3-41.8)	(27.4-46.5)	

Further analysis was applied using both univariate and multivariate analyses. The risk factors for EBV reactivation by univariate analysis are shown in **Table 3**. However, underlying diseases, and the presence of anti-HLA antibodies were not risk factors based on univariate analysis results. Upon multivariate analysis, the application of rituximab, cGVHD, CMV activation, and HLA-match were found to be independent predictive variables for the occurrence of EBV reactivation. The application of rituximab was proved to prevent the occurrence of EBV reactivation (HR 0.56, 95% CI 0.33-0.97, P=0.04, **Table 3**).

**Table 3:** Risk factors for EBV reactivation in entire patient cohort.

Variable	Univariate		Multivariate	
	Hazard Ratio (95%CI)	P value	Hazard Ratio (95%CI)	P value
Gender (Female vs. Male)	0.83 (0.51-1.33)	0.43		
Age (Continuous)	1.00 (0.98-1.02)	1		
Anti-HLA antibodies (Present vs. Not present)	0.839 (0.52-1.34)	0.46		
Rituximab (Yes vs. No)	0.28 (0.14-0.56)	3.50E-04	0.25 (0.12-0.51)	1.50E-04
Stem Cell Source (BM&PB vs. Others)	0.87 (0.53-1.43)	0.59		
aGVHD (Yes vs. No)	1.08 (0.67-1.75)	0.74		
cGVHD (Yes vs. No)	2.05 (1.28-3.28)	0.003	1.81 (1.12-2.93)	0.02
TMA (Yes vs. No)	0.88 (0.28-2.79)	0.83		
CMV (Yes vs. No)	2.49 (1.55-3.98)	1.50E-04	2.14 (1.32-3.46)	0.002
Lung Infection (Yes vs. No)	1.66 (1.04-2.66)	0.03	1.59 (0.99-2.53)	0.05
CR at HSCT (No vs. Yes)	0.76 (0.43-1.33)	0.34		
Conditioning (Others vs. MAC)	0.20 (0.03-1.44)	0.11		
Use of ATG (Yes vs. No)	1.37 (0.85-2.21)	0.19		
HLA Match (No vs. Yes)	2.82 (1.45-5.47)	0.002	2.99 (1.53-5.82)	0.001
Gender Match (No vs. Yes)	1.03 (0.64-1.65)	0.90		
Female to Male (Yes vs. No)	0.71 (0.31-1.64)	0.42		
ABO Match (No vs. Yes)	1.26 (0.79-2.02)	0.33		

### GVHD

**Figure 4:**
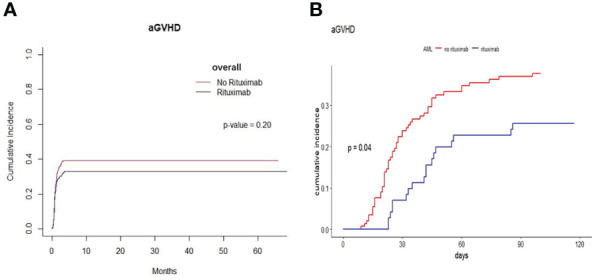
The cumulative incidence of aGVHD between rituximab and non-rituximab groups in different sets of populations. **(A)** The cumulative incidence of aGVHD in entire patient cohort. There was no difference in the cumulative incidence of aGVHD between rituximab and non-rituximab in entire patient cohort (P = 0.20). **(B)** The cumulative incidence of aGVHD in AML patients. There was comparable between 2 groups in the cumulative incidence of aGVHD in AML patients (P = 0.04).

We found that the cumulative incidence of aGVHD was 41.1% in the non-rituximab group and 34.4% in the rituximab group. The cumulative incidence of aGVHD was no significant difference between the two groups across the entire patient cohort (P=0.20, **Figure 4A**). Patients with AML took a vast majority in our cohort. In AML patients, the cumulative incidence of aGVHD was 37.7% in the non-rituximab group and 25.6% in the rituximab group. We found there was a difference in the cumulative incidence of aGVHD between rituximab and non-rituximab group (P=0.04, **Figure 4B**). However, in other underlying diseases, independent of the presence of anti-HLA antibodies, there was no difference in the cumulative incidence of aGVHD between non-rituximab and rituximab groups in these sets of groups (**Supplementary Figure S4**). We further explored the factors impacting the cumulative incidence of aGVHD in AML patients and found that rituximab prior to transplantation correlated with the decreased incidence of aGVHD in AML patients (HR 0.56 95%CI 0.33-0.97, P=0.037, **Table 4**).

**Table 4:** Risk factors for aGVHD in AML patients.

Characteristics	Univariate analysis		Multivariate analysis	
	P value	HR (95% CI for HR)	P value	HR (95% CI for HR)
age	0.66	1.00 (0.98-1.00)		
antibody	0.54	0.86 (0.54-1.40)		
Rituximab	0.04	0.57 (0.34-0.98)	0.04	0.56 (0.33-0.97)
Total MNC	0.29	1 .00(0.98-1.10)		
Total CD3	0.17	1.20 (0.93-1.50)	0.37	1.10 (0.85-1.50)
Total CD34+	0.10	0.89 (0.77-1.00)	0.09	0.88 (0.77-1.00)
nonCR	0.80	0.92 (0.47-1.80)		
Conditioning	0.82	0.79 (0.11-5.70)		
The usage of ATG	0.81	0.94 (0.59-1.50)		
HLAMatch	0.52	0.85 (0.52-1.40)		
SexMatch	0.64	1.10 (0.70-1.80)		
FtM	0.58	0.79 (0.34-1.80)		
ABOMatch	0.95	1.00 (0.63-1.60)		

The cumulative incidence of cGVHD across the entire cohort was determined to be 35.3%. There was not comparable in the cumulative incidence of cGVHD between non-rituximab and rituximab across the entire cohort (P=0.68), so as other sets of populations (**Supplementary Figure S5**).

### Other complications

TA-TMA was diagnosed in 25 of 520 patients in this study. Additionally, the incidence of TA-TMA was 5.11% in the rituximab group and 4.65% in the non-rituximab group. There was no significant difference between the two groups (*P*=0.830). Only 4 patients were diagnosed as VOD and there was not comparable between rituximab and non-rituximab groups (P=1).

### Prognosis

The cumulative incidence of relapse at 5 years was 12.0% and 9.2% in non-rituximab and rituximab groups across the entire patient cohort, respectively. Among those, there was not significant (P=0.33). There was no significant difference in the cumulative incidence of relapse in the rituximab group versus the non-rituximab group in all sets of groups (**Supplementary Figure S6**).

The NRM risk and PFS were not significantly different between the rituximab group and the non-rituximab group in entire cohort (**Supplementary Figure S7**).

The cumulative incidences of all measured OS in non-rituximab and rituximab groups were 73.1% and 77.2%, respectively, across the entire patient cohort. Among those, there was no significant difference (P=0.31). The OS was not significant between rituximab and non-rituximab groups in all sets of groups (**Supplementary Figure S8**).

In summary, we found there were no differences between rituximab and non-rituximab groups in the prognosis, suggesting that rituximab may not play a role in the prognosis of patients undergoing allo-HSCT.

## Discussion

To our knowledge, there were few articles about investigating the impact of rituximab administered before transplantation in allogeneic HSCT in a real-world study. In our study, there was no difference in the recovery of neutrophils and platelets in allo-HSCT patients between the rituximab and non-rituximab group. We found there was no impact on engraftment between the non-rituximab and rituximab groups when looking at only patients that were positive for anti-HLA antibodies. In previous studies, rituximab combination with the standard TBI/Cy transplant conditioning regimen for patients with CD20-positive acute lymphoblastic leukemia (ALL) and nonmyeloablative conditioning (fludarabine and cyclophosphamide) combination with rituximab for patients with relapsed follicular lymphoma were feasible and safe without delayed engraftment or added toxicity (32, 33). As we know, CD20 expression is not only confined to malignant cells but also normal B cells are also destroyed (9). Indeed, B lymphopoiesis is also targeted, and neutropenia can occur after repetitive use of rituximab (34, 35). In previous studies, patients treated with pre-emptive rituximab for EBV reactivation after allo-HSCT experienced neutropenia and a lower B cell reconstitution (35–37). However, in our study, the application of rituximab delayed the engraftment of platelets in the negative anti-HLA antibodies group. As rituximab was used to remove a subsets of B cells, it can subsequently affect the production of certain antibodies (38) and as a second line therapy for patients with idiopathic thrombocytopenic purpura (ITP) (38), a disease mediated by platelet antibodies that accelerate platelet destruction and inhibit the production of platelets (39).

We found that the application of rituximab decreased the occurrence of EBV reactivation in all patients. It is well established that EBV viremia is a common complication after allo-HSCT (40). Previous studies have reported that the incidence of EBV reactivation ranges from 0.6 to 26% (41, 42), with this being higher in the context of T-cell depletion (43, 44). There are a few studies about prophylactically eliminating B cells resulting in the prevention of EBV transmission to the recipient, due to the persistence of EBV requires the establishment of latent infection in recipient B cells (27, 34–37, 45–47). In a previous study, rituximab therapy within 6 months prior to allo-HSCT was also found to prevent EBV reactivation (47). In previous studies, severe aplastic anemia (SAA) patients undergoing unrelated donor transplantation received rituximab in combination with ATG-containing conditioning regimens to prevent EBV reactivation (27, 48). Previous studies showed that the risk factors for EBV activation were predominantly related to the degree of T cell depletion or impairment, including mismatch between donor and recipient, T cell depletion from graft, degree and duration of immunosuppression, and use of ATG or alemtuzumab (anti-CD52) (41, 45, 49–51). Furthermore, we found CMV activation, HLA mismatch and cGVHD were independent risk factors for EBV reactivation, while the usage of rituximab was proved to prevent EBV reactivation.

We also assessed the impact of rituximab on GVHD. The extent of B-cell involvement in the immunopathogenesis of aGVHD is unknown and elusive. B-cell dysfunction had been reported in earlier investigations on immune function in patients who had GVHD (4). Previous studies indicated that danger signals activate antigen presenting cells (APC) to efficiently present alloantigen to donor T cells and release cytokines (IL-12, IL-23, IL-6, IL-27, IL-10, TGF) which can expand and differentiate into pathogenic and regulatory donor T cells (52). However, regulatory B cells can minimize damage caused by aGVHD and reduce the risk of cGVHD development (35). They are distributed in IgM memory and transitional B-cell subsets (53). A study found that B cell reconstitution after pre-emptive rituximab administration favors the recovery of transitional and CD24+ CD38+ B cells that may exert regulatory functions (35). Moreover, data obtained in kidney transplant suggested that rituximab might be the depletion of locally active, intrarenal B cells, which act as immune stimulators and potent antigen presenting cells for T cells and macrophages in a rapid effect. In addition, depletion of local B cells inside the kidney could prevent the formation of intrarenal tertiary lymphoid structures that might otherwise lead to the development of chronic rejection (54).

Based on these concepts, in our study, we found rituximab had association with a reduced the occurrence of aGVHD in AML patients. The antigen presentation abilities of B cells might be abrogated in patients with lymphoma receiving rituximab within 6 months, resulting in a reduced risk of aGVHD (26). In a previous study, we explored the effect of rituximab at a single dose of 375 mg/m^2^, in combination with rATG, in the haplo-HSCT conditioning regimen of SAA in our center (55). Data indicated that B-cell depletion might also be effective for the prevention of aGVHD (55). In a previous study, the addition of rituximab (375mg/m^2^) intravenously on d-7, d-1, d7, and d14) to the standard Cy/TBI transplant conditioning regimen in ALL patients, the incidence of aGVHD was reduced when compared to reported GVHD rates in ALL patients (32). However, inhibition of antigen presentation by B cells with a rituximab-based conditioning regimen didn’t appear to reduce the incidence of aGVHD in allo-HSCT recipients in a retrospective cohort study (56). In this study, most patients were treated with reduced-intensity conditioning, while most patients with AML were given myeloablative regimen in our study.

There was no consensus on the effect of rituximab administration (before allo-HSCT or as part of conditioning regimens) on overall incidence of cGVHD (57).Some studies have indicated that the application of rituximab reduced the incidence of cGVHD. Since B cells can play a major role in the pathogenic process of cGVHD (58, 59), pre-emptive administration of rituximab could be helpful to reduce the occurrence of cGVHD. In a previous study, there was a trend towards a decreased incidence of cGVHD in rituximab patients receiving rituximab within 6 months (dose was not given) prior to reduced-intensity conditioning transplantation (RICT) (33).They also found that rituximab administration before transplantation eliminated B cells of recipients. Meanwhile, a work performed in a chronic GvHD model suggests that recipient B cell persistence may contribute to chronic GVHD. However, a retrospective study showed that patients who received rituximab within 6 months after allo-HCT had a lower incidence of severe cGVHD, but rituximab treatment didn’t affect the overall incidence of cGVHD (34, 57). Upon onset of cGVHD, the levels of rituximab present may not meet a therapeutic threshold and the recovery of the donor B-cells, T-cells, and other antigen-presenting cells (APCs) might have played a significant role in the initiation and maintenance of cGVHD (4, 33, 57, 60). Besides, repetitive administration of rituximab could lead to neutropenia, which may can’t meet the threshold to the occurrence of cGVHD. And the lack of impact on cGVHD in our study could be explained by the timing and dose of the application of rituximab (26).

Recently, the main purpose of pre-emptive rituximab was considered to optimize the cytoreductive capability of the conditioning regimens (61). A previous randomized study has compared preparative regimens with or without rituximab in autologous stem cell transplantation setting (4). They found that, according to multivariate analysis, there was a significant improvement in the failure-free survival (FFS) rate and OS rate in rituximab-treated patients. The antileukemic effect of HSCT was related to the cytoreductive effect of the preparative regimen (32). In previous studies, there was no significant increase to the risk of relapse in rituximab treatment groups. In terms of outcomes, rituximab didn’t negatively impact the PFS and OS rates at two years among patients receiving rituximab treatment (24, 33). In a previous study, data indicated that inclusion of rituximab in SAA conditioning regimens might enhance disease control, particularly in non-myeloablative settings (48). These results were consistent with our study. Given that this study was retrospective and used patients from only one center, further studies are needed to define the optimal dose and timing of rituximab in allo-HSCT. Since this was a real-world study, we relied on the experience of clinicians. The long-term effects of rituximab therapy in the context of HSCT are unknown and there is a lack of data on the reconstitution of B cells. In conclusion, the application of rituximab more than once two weeks prior to transplantation reduced the incidence of EBV reactivation across the entire patient cohort in our study. And there were significant differences in other outcomes. We then explored the impact of rituximab on more specific patient groups within our cohort. Considering the role of anti-HLA antibodies in allo-HSCT patient outcomes, we found that the application of rituximab delayed the engraftment of platelets in the negative anti-HLA antibodies group. We found that the application of rituximab may reduce aGVHD in male and AML patients. Importantly, our study indicated that the application of rituximab prior to transplantation might decrease EBV reactivation through deleting previously infected B cells in circulation.

## Data availability statement

The original contributions presented in the study are included in the article/**Supplementary Material**. Further inquiries can be directed to the corresponding authors.

## Ethics statement

The studies involving human participants were reviewed and approved by the First Affiliated Hospital of Soochow University. Written informed consent to participate in this study was provided by the participants’ legal guardian/next of kin. Written informed consent was obtained from the individual(s), and minor(s)’ legal guardian/next of kin, for the publication of any potentially identifiable images or data included in this article.

## Author contributions

XW and DW designed the study. RJ, HL, YZ, HW, LL, SZ, XM, ZT, and JH performed the research and collected the data. XWe and YX wrote the paper and analyzed the data. All authors contributed to the article and approved the submitted version.

## Funding

This work was supported by the National Natural Science Foundation of China (Grant Nos. 81974001 and 82170222); the Jiangsu Natural Science Foundation (BK20211070); The Key Disease Program of Suzhou (LCZX202101); National Science and Technology Major Project (2017ZX09304021); National Key R&D Program of China (2019YFC0840604, 2017YFA0104502); Priority Academic Program Development of Jiangsu Higher Education Institutions (PAPD); Jiangsu Medical Outstanding Talents Project (JCRCA2016002); and Jiangsu Provincial Key Research and Development Program (BE2019656); Jiangsu Provincial Key Medical Center (YXZXA2016002).

## Acknowledgments

The authors would like to thank the reviewers for their detailed comments and feedback that assisted in the revising of our original manuscript.

## Conflict of interest

The authors declare that the research was conducted in the absence of any commercial or financial relationships that could be construed as a potential conflict of interest.

## Publisher’s note

All claims expressed in this article are solely those of the authors and do not necessarily represent those of their affiliated organizations, or those of the publisher, the editors and the reviewers. Any product that may be evaluated in this article, or claim that may be made by its manufacturer, is not guaranteed or endorsed by the publisher.

## References

[1] Edwards JC Szczepanski L Szechinski J Filipowicz-Sosnowska A Emery P Close DR . Efficacy of b-cell-targeted therapy with rituximab in patients with rheumatoid arthritis. N Engl J Med (2004) 350(25):2572–81. doi: 10.1056/NEJMoa032534 15201414

[2] Pescovitz MD . Rituximab, an anti-cd20 monoclonal antibody: history and mechanism of action. Am J Transplant (2006) 6(5 Pt 1):859–66. doi: 10.1111/j.1600-6143.2006.01288.x 16611321

[3] Macklin PS Morris PJ Knight SR . A systematic review of the use of rituximab for desensitization in renal transplantation. Transplantation (2014) 98(8):794–805. doi: 10.1097/TP.0000000000000362 25321163

[4] Ratanatharathorn V Pavletic S Uberti JP . Clinical applications of rituximab in allogeneic stem cell transplantation: anti-tumor and immunomodulatory effects. Cancer Treat Rev (2009) 35(8):653–61. doi: 10.1016/j.ctrv.2009.07.004 19682801

[5] Cerny T Borisch B Introna M Johnson P Rose AL . Mechanism of action of rituximab. Anticancer Drugs (2002) 13(Suppl 2):S3–10. doi: 10.1097/00001813-200211002-00002 12710585

[6] Rougé L Chiang N Steffek M Kugel C Croll TI Tam C . Structure of CD20 in complex with the therapeutic monoclonal antibody rituximab. Science (2020) 367(6483):1224–30. doi: 10.1126/science.aaz9356 32079680

[7] Marshall MJE Stopforth RJ Cragg MS . Therapeutic antibodies: What have we learnt from targeting CD20 and where are we going? Front Immunol (2017) 8:1245. doi: 10.3389/fimmu.2017.01245 29046676 PMC5632755

[8] Cragg MS Glennie MJ . Antibody specificity controls in vivo effector mechanisms of anti-CD20 reagents. Blood (2004) 103(7):2738–43. doi: 10.1182/blood-2003-06-2031 14551143

[9] Salles G Barrett M Foa R Maurer J O'Brien S Valente N . Rituximab in b-cell hematologic malignancies: A review of 20 years of clinical experience. Adv Ther (2017) 34(10):2232–73. doi: 10.1007/s12325-017-0612-x PMC5656728 28983798

[10] Jazirehi AR Bonavida B . Cellular and molecular signal transduction pathways modulated by rituximab (rituxan, anti-CD20 mAb) in non-hodgkin's lymphoma: implications in chemosensitization and therapeutic intervention. Oncogene (2005) 24(13):2121–43. doi: 10.1038/sj.onc.1208349 15789036

[11] Berghen N Vulsteke JB Westhovens R Lenaerts J De Langhe E . Rituximab in systemic autoimmune rheumatic diseases: indications and practical use. Acta Clin Belg (2019) 74(4):272–9. doi: 10.1080/17843286.2018.1521904 30253707

[12] Frampton JE . Rituximab: A review in pemphigus vulgaris. Am J Clin Dermatol (2020) 21(1):149–56. doi: 10.1007/s40257-019-00497-9 31838645

[13] Tandan R Hehir MK 2nd Waheed W Howard DB . Rituximab treatment of myasthenia gravis: A systematic review. Muscle Nerve (2017) 56(2):185–96. doi: 10.1002/mus.25597 28164324

[14] Marfo K Lu A Ling M Akalin E . Desensitization protocols and their outcome. Clin J Am Soc Nephrol (2011) 6(4):922–36. doi: 10.2215/CJN.08140910 21441131

[15] Zachary AA Lucas DP Montgomery RA Leffell MS . Rituximab prevents an anamnestic response in patients with cryptic sensitization to HLA. Transplantation (2013) 95(5):701–4. doi: 10.1097/TP.0b013e31827be3c1 23503502

[16] Surendra M Raju SB Raju N Chandragiri S Mukku KK Uppin MS . Rituximab in the treatment of refractory late acute antibody-mediated rejection: Our initial experience. Indian J Nephrol (2016) 26(5):317–21. doi: 10.4103/0971-4065.177207 PMC5015507 27795623

[17] Moreso F Crespo M Ruiz JC Torres A Gutierrez-Dalmau A Osuna A . Treatment of chronic antibody mediated rejection with intravenous immunoglobulins and rituximab: A multicenter, prospective, randomized, double-blind clinical trial. Am J Transplant (2018) 18(4):927–35. doi: 10.1111/ajt.14520 28949089

[18] Arai S Miklos DB . Rituximab in hematopoietic cell transplantation. Expert Opin Biol Ther (2010) 10(6):971–82. doi: 10.1517/14712598.2010.485982 20420511

[19] Leto Barone AA Sun Z Montgomery RA Lee WP Brandacher G . Impact of donor-specific antibodies in reconstructive transplantation. Expert Rev Clin Immunol (2013) 9(9):835–44. doi: 10.1586/1744666X.2013.824667 24070047

[20] Kharfan-Dabaja MA Nishihori T Otrock ZK Haidar N Mohty M Hamadani M . Monoclonal antibodies in conditioning regimens for hematopoietic cell transplantation. Biol Blood Marrow Transpl (2013) 19(9):1288–300. doi: 10.1016/j.bbmt.2013.04.016 23618718

[21] V Villarreal-González R E de Lira-Quezada C N González-Díaz S N González-Díaz S González-Llano O . Rituximab desensitization in pediatric acute lymphoblastic leukemia with severe anaphylaxis. J Oncol Pharm Pract (2020) 27(3):747–50. doi: 10.1177/1078155220948596 32787558

[22] Ciurea SO Al Malki MM Kongtim P Zou J Aung FM Rondon G . Treatment of allosensitized patients receiving allogeneic transplantation. Blood Adv (2021) 5(20):4031–43. doi: 10.1182/bloodadvances.2021004862 PMC8945639 34474478

[23] Puliyanda DP Jordan SC Kim IK Patel M Murthy A Huang E . Use of rituximab for persistent EBV DNAemia, and its effect on donor-specific antibody development in pediatric renal transplant recipients: A case series. Pediatr Transplant (2021) 25(8):e14113. doi: 10.1111/petr.14113 34418254

[24] van Dorp S Pietersma F Wolfl M Verdonck LF Petersen EJ Lokhorst HM . Rituximab treatment before reduced-intensity conditioning transplantation associates with a decreased incidence of extensive chronic GVHD. Biol Blood Marrow Transpl (2009) 15(6):671–8. doi: 10.1016/j.bbmt.2009.02.005 19450751

[25] Jing S Song Y Song J Pang S Quan C Zhou L . Responsiveness to low-dose rituximab in refractory generalized myasthenia gravis. J Neuroimmunol (2017) 311:14–21. doi: 10.1016/j.jneuroim.2017.05.021 28789841

[26] Crocchiolo R Castagna L El-Cheikh J Helvig A Furst S Faucher C . Prior rituximab administration is associated with reduced rate of acute GVHD after *in vivo* T-cell depleted transplantation in lymphoma patients. Exp Hematol (2011) 39(9):892–6. doi: 10.1016/j.exphem.2011.06.006 21703987

[27] Dominietto A Tedone E Soracco M Bruno B Raiola AM Van Lint MT . *In vivo* b-cell depletion with rituximab for alternative donor hemopoietic SCT. Bone Marrow Transplant (2012) 47(1):101–6. doi: 10.1038/bmt.2011.28 21460867

[28] Bordon V Padalko E Benoit Y Dhooge C Laureys G . Incidence, kinetics, and risk factors of Epstein-Barr virus viremia in pediatric patients after allogeneic stem cell transplantation. Pediatr Transpl (2012) 16(2):144–50. doi: 10.1111/j.1399-3046.2011.01634.x 22288846

[29] Ciurea SO Cao K Fernandez-Vina M Kongtim P Malki MA Fuchs E . The European society for blood and marrow transplantation (EBMT) consensus guidelines for the detection and treatment of donor-specific anti-HLA antibodies (DSA) in haploidentical hematopoietic cell transplantation. Bone Marrow Transplant (2018) 53(5):521–34. doi: 10.1038/s41409-017-0062-8 PMC7232774 29335625

[30] Zhang R He Y Yang D Jiang E Ma Q Pang A . Combination treatment of rituximab and donor platelets infusion to reduce donor-specific anti-HLA antibodies for stem cells engraftment in haploidentical transplantation. J Clin Lab Anal (2020) 34(7):e23261. doi: 10.1002/jcla.23261 32112480 PMC7370703

[31] Jianrong G Xiya W Yanting G Yiyu X Huiyu L Shiyuan Z . Relationship of oropharyngeal colonization microorganisms to clinical outcomes within 100 days after allogeneic hematopoietic stem cell transplantation. Transplant Cell Ther (2022) 28(8):496.e1–.e7. doi: 10.1016/j.jtct.2022.05.017 35589057

[32] Kebriaei P Saliba RM Ma C Ippoliti C Couriel DR de Lima M . Allogeneic hematopoietic stem cell transplantation after rituximab-containing myeloablative preparative regimen for acute lymphoblastic leukemia. Bone Marrow Transplant (2006) 38(3):203–9. doi: 10.1038/sj.bmt.1705425 16799614

[33] Khouri IF McLaughlin P Saliba RM Hosing C Korbling M Lee MS . Eight-year experience with allogeneic stem cell transplantation for relapsed follicular lymphoma after nonmyeloablative conditioning with fludarabine, cyclophosphamide, and rituximab. Blood (2008) 111(12):5530–6. doi: 10.1182/blood-2008-01-136242 PMC4624452 18411419

[34] van der Velden WJ Mori T Stevens WB de Haan AF Stelma FF Blijlevens NM . Reduced PTLD-related mortality in patients experiencing EBV infection following allo-SCT after the introduction of a protocol incorporating pre-emptive rituximab. Bone Marrow Transpl (2013) 48(11):1465–71. doi: 10.1038/bmt.2013.84 23749107

[35] Stocker N Labopin M Boussen I Paccoud O Bonnin A Malard F . Pre-emptive rituximab treatment for Epstein-Barr virus reactivation after allogeneic hematopoietic stem cell transplantation is a worthwhile strategy in high-risk recipients: a comparative study for immune recovery and clinical outcomes. Bone Marrow Transplant (2020) 55(3):586–94. doi: 10.1038/s41409-019-0699-6 31562397

[36] Petropoulou AD Porcher R Peffault de Latour R Xhaard A Weisdorf D Ribaud P . Increased infection rate after preemptive rituximab treatment for Epstein-Barr virus reactivation after allogeneic hematopoietic stem-cell transplantation. Transplantation (2012) 94(8):879–83. doi: 10.1097/TP.0b013e3182664042 23001354

[37] McIver Z Stephens N Grim A Barrett AJ . Rituximab administration within 6 months of T cell-depleted allogeneic SCT is associated with prolonged life-threatening cytopenias. Biol Blood Marrow Transplant (2010) 16(11):1549–56. doi: 10.1016/j.bbmt.2010.05.004 PMC2947610 20580848

[38] Arnold DM Vrbensky JR Karim N Smith JW Liu Y Ivetic N . The effect of rituximab on anti-platelet autoantibody levels in patients with immune thrombocytopenia. Br J Haematol (2017) 178(2):302–7. doi: 10.1111/bjh.14664 28444742

[39] Lucchini E Zaja F Bussel J . Rituximab in the treatment of immune thrombocytopenia: what is the role of this agent in 2019? Haematologica (2019) 104(6):1124–35. doi: 10.3324/haematol.2019.218883 PMC6545833 31126963

[40] Tangye SG Latour S . Primary immunodeficiencies reveal the molecular requirements for effective host defense against EBV infection. Blood (2020) 135:644–55. doi: 10.1182/blood.2019000928 31942615

[41] Wang H Zhang TT Qi JQ Chu TT Miao M Qiu HY . Incidence, risk factors, and clinical significance of Epstein-Barr virus reactivation in myelodysplastic syndrome after allogeneic haematopoietic stem cell transplantation. Ann Hematol (2019) 98(4):987–96. doi: 10.1007/s00277-019-03603-3 30715567

[42] Styczynski J Tridello G Gil L Ljungman P Mikulska M Ward KN . Prognostic impact of EBV serostatus in patients with lymphomas or chronic malignancies undergoing allogeneic HCT. Bone Marrow Transplant (2019) 54(12):2060–71. doi: 10.1038/s41409-019-0627-9 31363166

[43] Raberahona M Wackenheim C Germi R Carre M Bulabois CE Thiebaut A . Dynamics of Epstein-Barr viral load after hematopoietic stem cell transplantation and effect of preemptive rituximab therapy. Transpl Infect Dis (2016) 18(6):889–95. doi: 10.1111/tid.12618 27696681

[44] Huang XJ Liu DH Liu KY Xu LP Chen H Han W . Haploidentical hematopoietic stem cell transplantation without *in vitro* T-cell depletion for the treatment of hematological malignancies. Bone Marrow Transplant (2006) 38(4):291–7. doi: 10.1038/sj.bmt.1705445 16883312

[45] Dumas PY Ruggeri A Robin M Crotta A Abraham J Forcade E . Incidence and risk factors of EBV reactivation after unrelated cord blood transplantation: a eurocord and societe francaise de greffe de moelle-therapie cellulaire collaborative study. Bone Marrow Transplant (2013) 48(2):253–6. doi: 10.1038/bmt.2012.117 22773124

[46] Burns DM Rana S Martin E Nagra S Ward J Osman H . Greatly reduced risk of EBV reactivation in rituximab-experienced recipients of alemtuzumab-conditioned allogeneic HSCT. Bone Marrow Transpl (2016) 51(6):825–32. doi: 10.1038/bmt.2016.19 PMC4880046 26901708

[47] Van Besien K Bachier-Rodriguez L Satlin M Brown MA Gergis U Guarneri D . Prophylactic rituximab prevents EBV PTLD in haplo-cord transplant recipients at high risk. Leuk Lymphoma (2019) 60(7):1693–6. doi: 10.1080/10428194.2018.1543877 30741059

[48] Gatwood KS Culos KA Binari LA Engelhardt BG Kassim A Byrne MT . Outcomes of a novel rituximab-based non-myeloablative conditioning regimen for hematopoietic cell transplantation in severe aplastic anemia. Bone Marrow Transplant (2018) 53(6):795–9. doi: 10.1038/s41409-018-0124-6 29540851

[49] Scheinberg P Fischer SH Li L Nunez O Wu CO Sloand EM . Distinct EBV and CMV reactivation patterns following antibody-based immunosuppressive regimens in patients with severe aplastic anemia. Blood (2007) 109(8):3219–24. doi: 10.1182/blood-2006-09-045625 PMC1852232 17148582

[50] Delapierre B Reman O Dina J Breuil C Bellal M Johnson-Ansah H . Low dose rituximab for pre-emptive treatment of Epstein Barr virus reactivation after allogenic hematopoietic stem cell transplantation. Curr Res Transl Med (2019) 67(4):145–8. doi: 10.1016/j.retram.2019.03.001 30871955

[51] Park SS Cho SY Han E Min GJ Park S Yoon JH . Reactivation and dynamics of cytomegalovirus and Epstein-Barr virus after rabbit antithymocyte globulin and cyclosporine for aplastic anemia. Eur J Haematol (2019) 103(4):433–41. doi: 10.1111/ejh.13308 31381187

[52] Hill GR Koyama M . Cytokines and Co-stimulation in acute graft-versus-Host disease. Blood (2020) 136(4):418–28. doi: 10.1182/blood.2019000952 32526028 PMC7378458

[53] De Masson A Socié G Bagot M Bensussan A Bouaziz J-D . Deficient regulatory b cells in human chronic graft-versus-host disease. OncoImmunology (2015) 4:e1016707. doi: 10.1080/2162402x.2015.1016707 26140245 PMC4485800

[54] Steinmetz OM Lange-Husken F Turner JE Vernauer A Helmchen U Stahl RA . Rituximab removes intrarenal b cell clusters in patients with renal vascular allograft rejection. Transplantation (2007) 84(7):842–50. doi: 10.1097/01.tp.0000282786.58754.2b 17984836

[55] Liu L Zhang Y Liu S Zhou H Wang Q Qiu H . Outcomes of conditioning with rabbit antithymocyte globulin and rituximab in haploidentical haematopoietic stem cell transplantation in patients with severe aplastic anaemia. Bone Marrow Transplant (2020) 55(9):1854–6. doi: 10.1038/s41409-020-0788-6 31959891

[56] Marini BL Markstrom D Frame D . Risk of graft-versus-host disease with rituximab-containing conditioning regimens in allogeneic hematopoietic stem cell transplant. J Oncol Pharm Pract (2017) 23(4):255–63. doi: 10.1177/1078155216637216 26970572

[57] Kharfan-Dabaja MA Cutler CS . Rituximab for prevention and treatment of graft-versus-host disease. Int J Hematol (2011) 93(5):578–85. doi: 10.1007/s12185-011-0855-2 21547615

[58] Kapur R Ebeling S Hagenbeek A . B-cell involvement in chronic graft-versus-host disease. Haematologica (2008) 93(11):1702–11. doi: 10.3324/haematol.13311 18728020

[59] Zeiser R Sarantopoulos S Blazar BR . B-cell targeting in chronic graft-versus-host disease. Blood (2018) 131(13):1399–405. doi: 10.1182/blood-2017-11-784017 PMC6031308 29437591

[60] Masjosthusmann K Ehlert K Eing BR Roth J Koehler G Juergens H . Delay in b-lymphocyte recovery and function following rituximab for EBV-associated lymphoproliferative disease early post-allogeneic hematopoietic SCT. Bone Marrow Transplant (2009) 43(9):679–84. doi: 10.1038/bmt.2008.385 19029962

[61] Marks R Potthoff K Hahn J Ihorst G Bertz H Spyridonidis A . Reduced-toxicity conditioning with fludarabine, BCNU, and melphalan in allogeneic hematopoietic cell transplantation: particular activity against advanced hematologic malignancies. Blood (2008) 112(2):415–25. doi: 10.1182/blood-2007-08-104745 18451310

