# Supplementary figures and images for: The impact of Rituximab administered before transplantation in patients undergoing allogeneic hematopoietic stem cell transplantation: A real-world study
Source: Front Immunol. 2022 Aug 31;13:967026. doi: 10.3389/fimmu.2022.967026 (PMC9471377; doi:10.3389/fimmu.2022.967026)

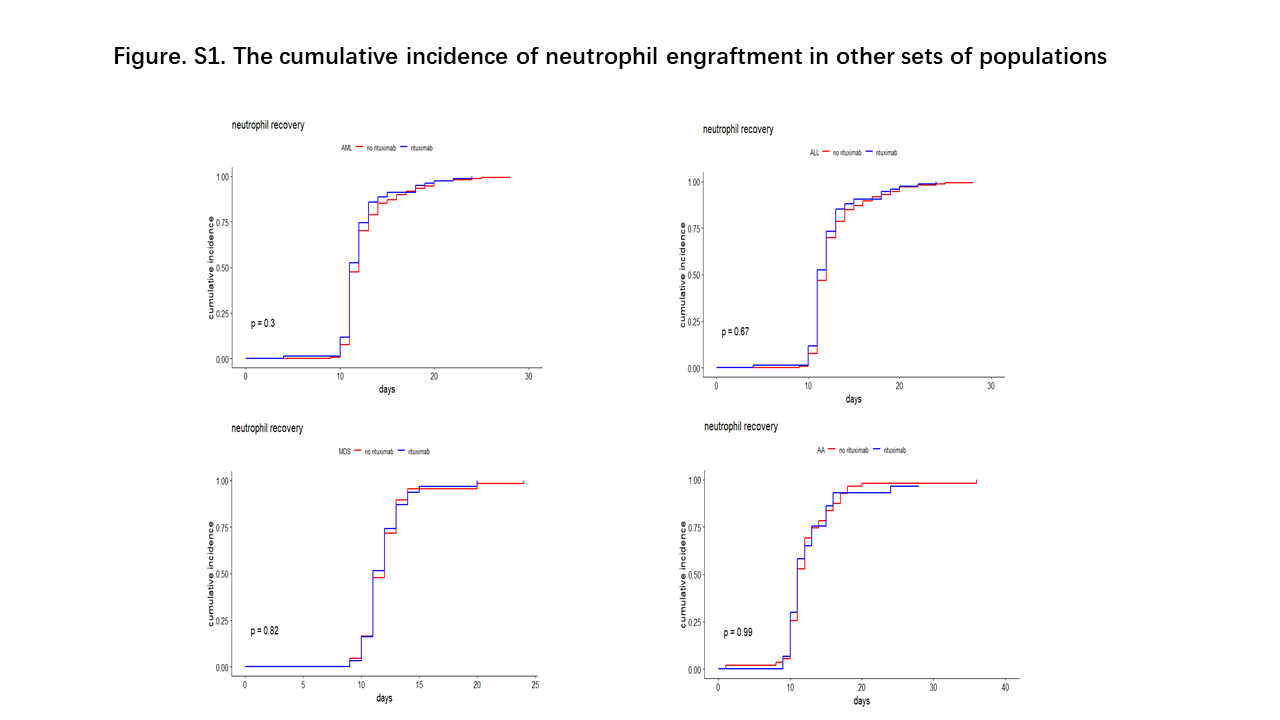

Supplement: Supplementary file 1 [file DataSheet_1.zip › Supplementary Information/Figure.S1.TIF]

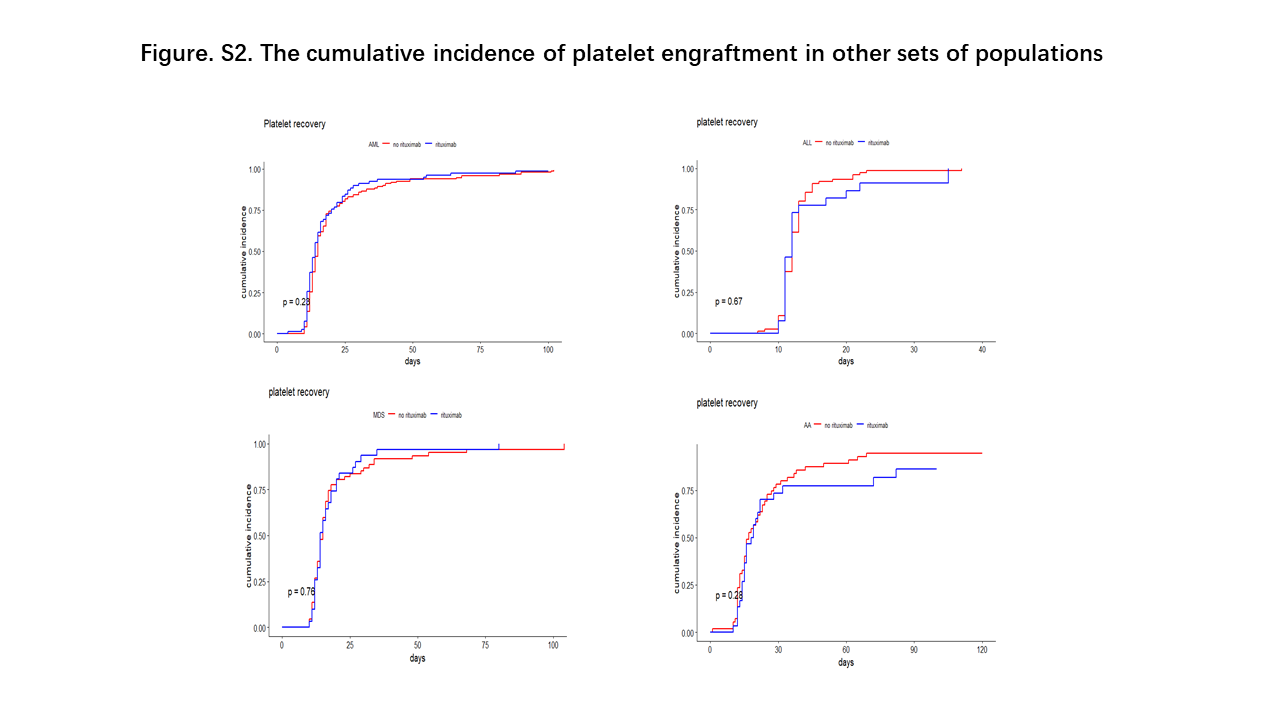

Supplement: Supplementary file 1 [file DataSheet_1.zip › Supplementary Information/Figure.S2.TIF]

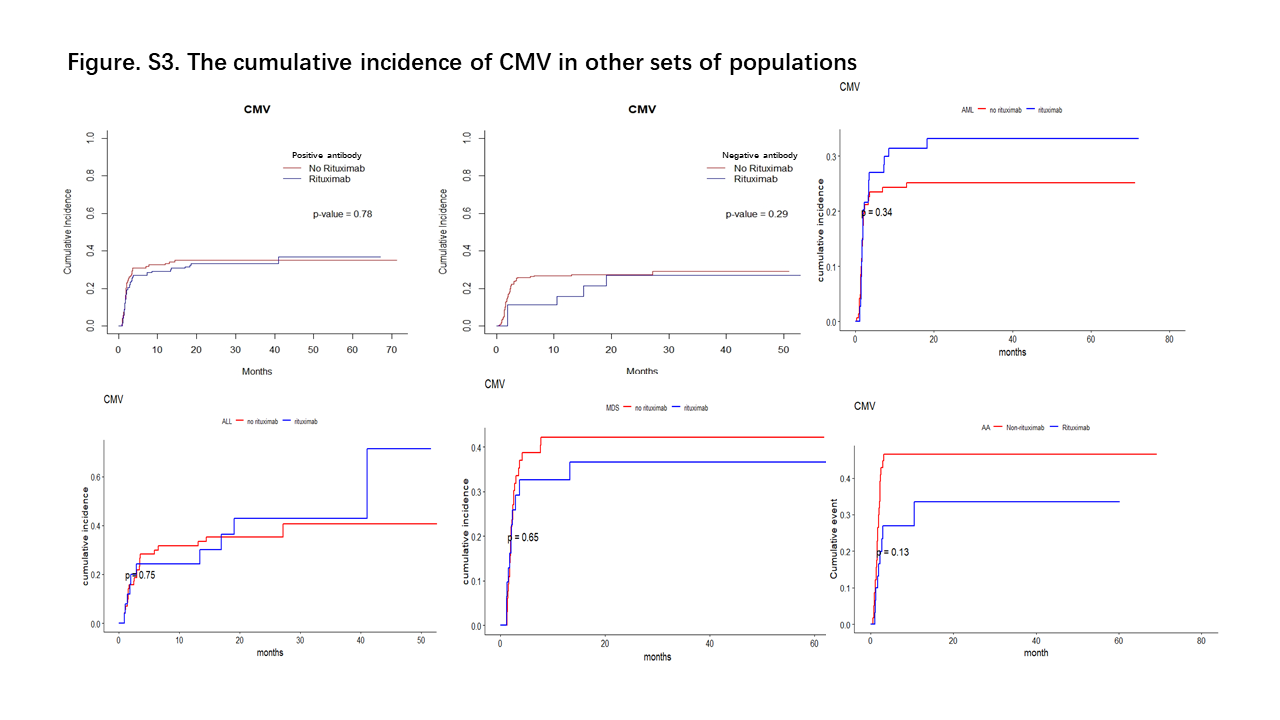

Supplement: Supplementary file 1 [file DataSheet_1.zip › Supplementary Information/Figure.S3.TIF]

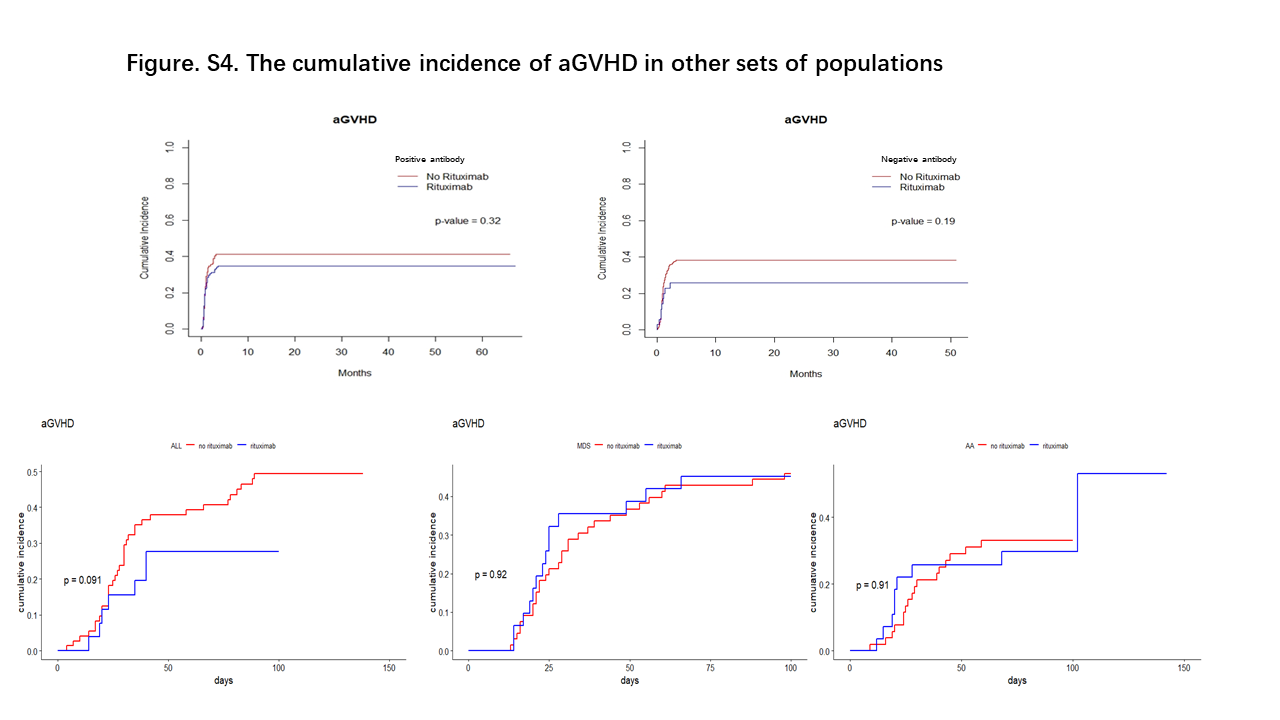

Supplement: Supplementary file 1 [file DataSheet_1.zip › Supplementary Information/Figure.S4.TIF]

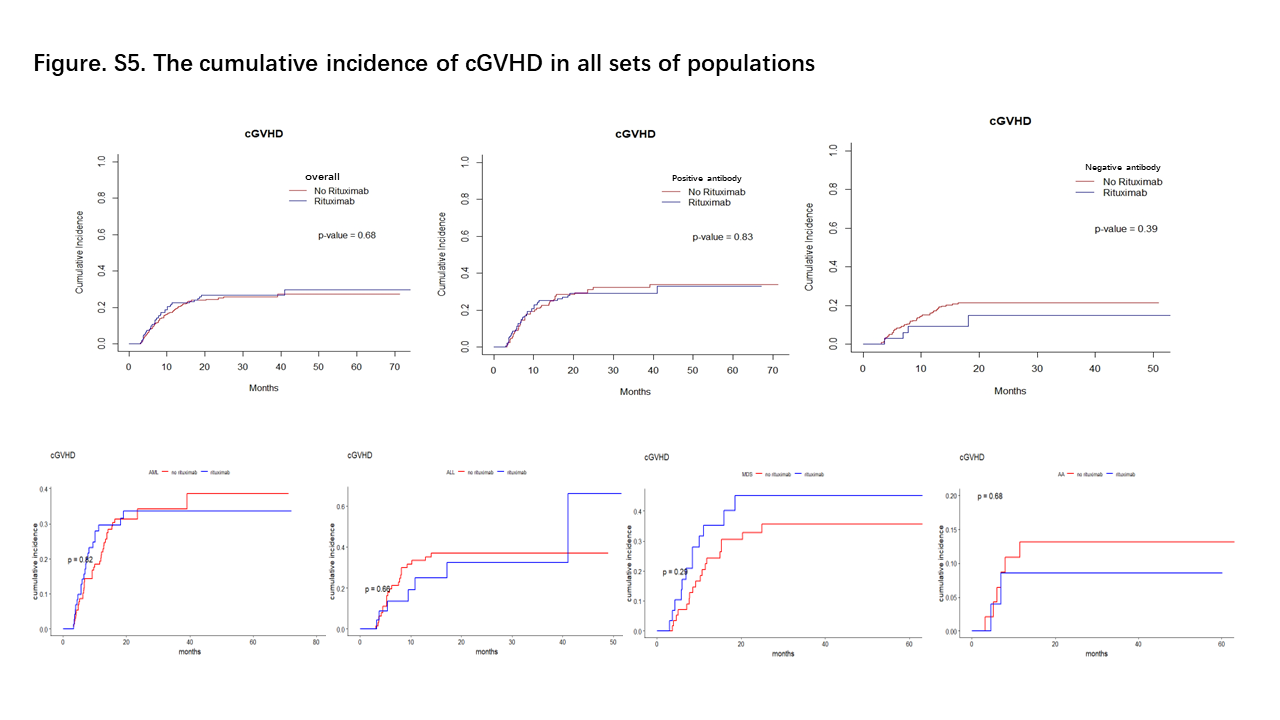

Supplement: Supplementary file 1 [file DataSheet_1.zip › Supplementary Information/Figure.S5.TIF]

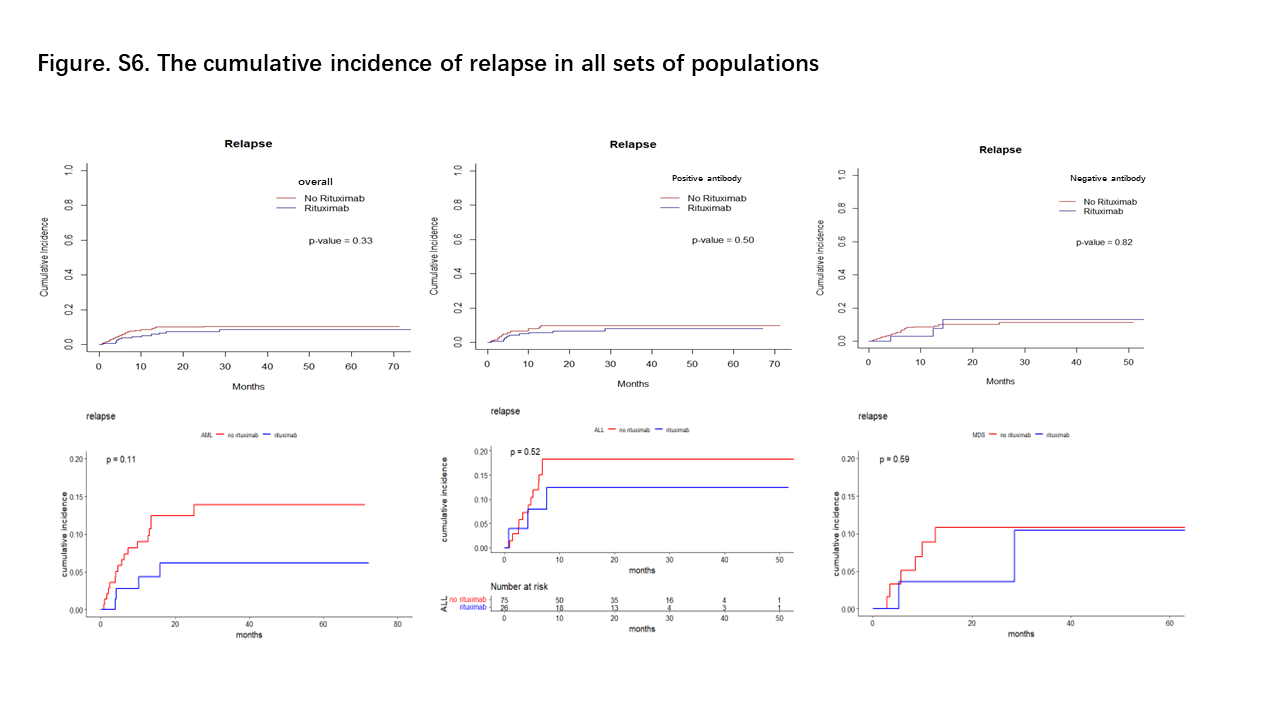

Supplement: Supplementary file 1 [file DataSheet_1.zip › Supplementary Information/Figure.S6.TIF]

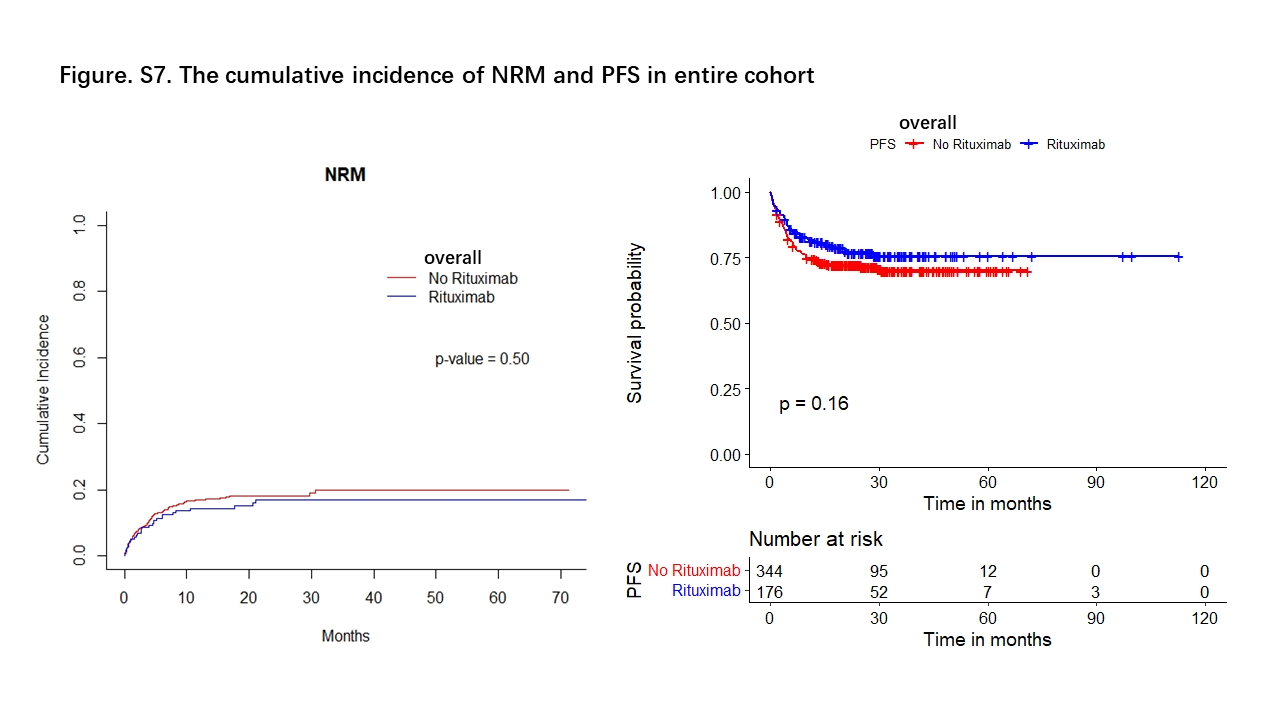

Supplement: Supplementary file 1 [file DataSheet_1.zip › Supplementary Information/Figure.S7.TIF]

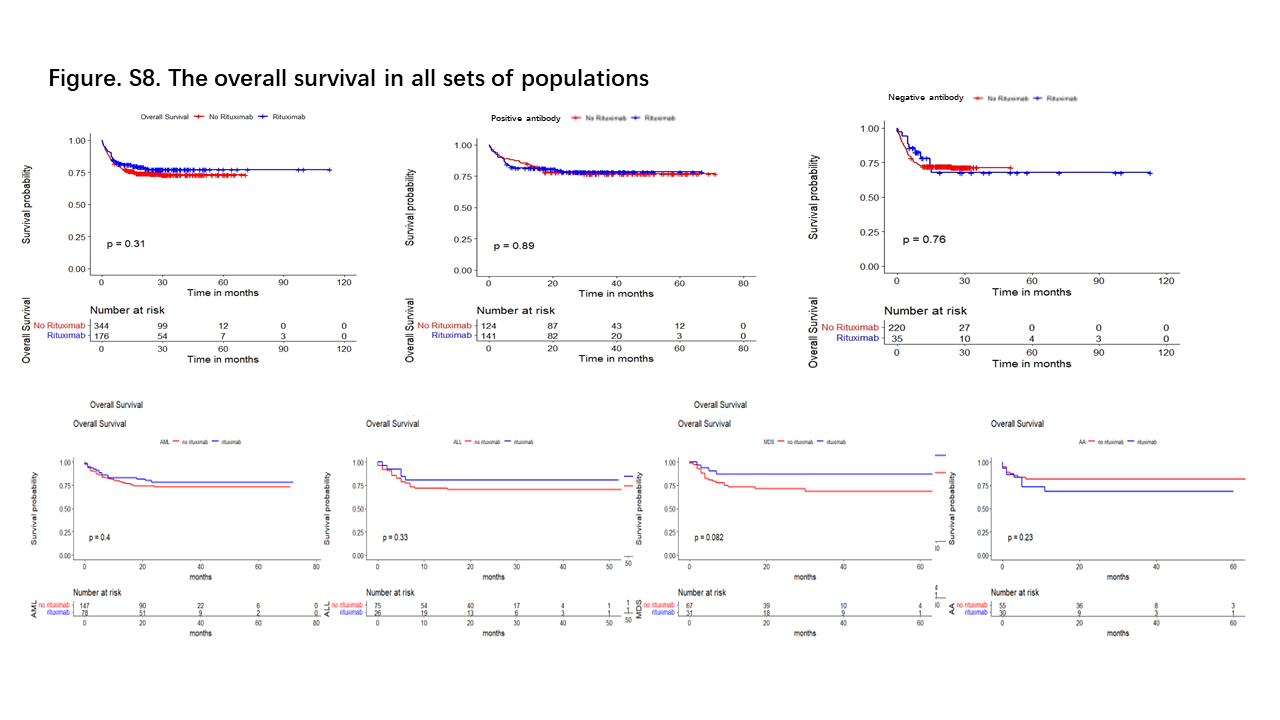

Supplement: Supplementary file 1 [file DataSheet_1.zip › Supplementary Information/Figure.S8.TIF]
